# Extracting and connecting chemical structures from text sources using chemicalize.org

**DOI:** 10.1186/1758-2946-5-20

**Published:** 2013-04-23

**Authors:** Christopher Southan, Andras Stracz

**Affiliations:** 1 TW2Informatics Ltd, Göteborg, 42166, Sweden; 2 ChemAxon Kft, Budapest, 1031, Hungary

## Abstract

**Background:**

Exploring bioactive chemistry requires navigating between structures and data from a variety of text-based sources. While PubChem currently includes approximately 16 million document-extracted structures (15 million from patents) the extent of public inter-document and document-to-database links is still well below any estimated total, especially for journal articles. A major expansion in access to text-entombed chemistry is enabled by chemicalize.org. This on-line resource can process IUPAC names, SMILES, InChI strings, CAS numbers and drug names from pasted text, PDFs or URLs to generate structures, calculate properties and launch searches. Here, we explore its utility for answering questions related to chemical structures in documents and where these overlap with database records. These aspects are illustrated using a common theme of Dipeptidyl Peptidase 4 (DPPIV) inhibitors.

**Results:**

Full-text open URL sources facilitated the download of over 1400 structures from a DPPIV patent and the alignment of specific examples with IC50 data. Uploading the SMILES to PubChem revealed extensive linking to patents and papers, including prior submissions from chemicalize.org as submitting source. A DPPIV medicinal chemistry paper was completely extracted and structures were aligned to the activity results table, as well as linked to other documents via PubChem. In both cases, key structures with data were partitioned from common chemistry by dividing them into individual new PDFs for conversion. Over 500 structures were also extracted from a batch of PubMed abstracts related to DPPIV inhibition. The drug structures could be stepped through each text occurrence and included some converted MeSH-only IUPAC names not linked in PubChem. Performing set intersections proved effective for detecting compounds-in-common between documents and merged extractions.

**Conclusion:**

This work demonstrates the utility of chemicalize.org for the exploration of chemical structure connectivity between documents and databases, including structure searches in PubChem, InChIKey searches in Google and the chemicalize.org archive. It has the flexibility to extract text from any internal, external or Web source. It synergizes with other open tools and the application is undergoing continued development. It should thus facilitate progress in medicinal chemistry, chemical biology and other bioactive chemistry domains.

## Background

The majority of chemical information and related data generated by biomedical research is specified in text form [1]. A proportion of these primary reports have been captured in public and commercial databases that include a document cross-reference linked to standard chemical representations [2,3]. Two basic methods are used to populate chemical databases via text. The first is expert manual curation (EMC) typically using a chemical sketcher for input. The second is automated name-to-structure conversion, also termed chemical named entity recognition (CNER). A third option, automated conversion of images to structures, has only just begun to contribute to public database entries via SureChemOpen [4].

A number of questions arise in regard to the global corpus of bioactive chemistry represented in text. These include (a) the total “out there” (b) the number represented in major public databases and (c) the ratio between source types. The upper limit for (a) could be the 70 million substances collated in the CAS commercial database but there are factors suggesting this exceeds the text-based corpus [5]. At 47 million, PubChem is not only the largest open repository but also provides content counts by submission types that can be used to answer (b) and (c) [6]. Patent-extracted structures have four major sources in PubChem. Three of these use CNER, SureChem (9.3 million) SCRIPDB (4.0 million) and IBM (2.4 million). The fourth, Thomson Pharma, is an EMC source (3.8 million). The union between these is 15 million. The largest journal extraction source is ChEMBL, with 0.8 million structures, and PubMed abstracts have 0.2 million linked structures. The chemistry capture ratio for patents: papers: abstracts is therefore approximately 70:4:1, with the union being 16 million. Even if the 70 million CAS-substances exceeded the text-specified total, the implication is that explicit document links for anywhere between 20 and 40 million unique structures are missing from public databases. Paradoxically, because of access constraints, this shortfall is largest for journal content, since the availability of full-text from the major patent offices is now largely complete [7].

Researchers exploring bioactive chemistry thus need ways of extracting structures from document “tombs”. In this work we explore the utility of chemicalize.org for this task [8]. Developed by ChemAxon, this web application uses a CNER algorithm and dictionaries to identify chemical structures in text from names and identifiers. The value of this lies in addressing practical questions, the answers to which are important to support decisions in both academic and commercial R&D settings.

## Implementation

### The chemicalize.org interface

**Figure 1:**
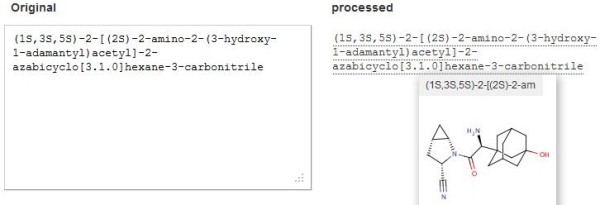
**Home page.** The IUPAC name for saxyglyptin (from PubChem CID 11243969) has been pasted in the query box on the left. This has been processed on the right as indicated by complete dotted underlining. Moving the mouse over this dotted line renders the structure (inset box on the right) and provides access to a full set of links.

The operational details of chemicalize.org have been recently reviewed [9]. There is also support material associated with the web site, including slide sets, videos and a technical update blog. A brief introduction is provided below, staring with the main page (Figure 1).

The application has four principle inputs, text strings, a sketcher interface, URLs and PDF uploads. For all of these, chemical entities in the text will be converted if they are recognized as semantic names, CAS numbers, IUPAC names, SMILES or InChI strings. The conversions automatically generate the quartet of IUPAC, SMILES, InChI string and the InChIKey, together with ancillary identifiers. For a web page or PDF, structures found are displayed as a ribbon of images at the top of the page. These present the conversions in order of first occurrence in the document, accompanied in brackets by the count of occurrences of that structure within the document, and, if a second bracket is included, the number of different names that structure had in the document.

### Sources and downloads

Document retrieval is outside the scope of this report but we can indicate compatible sources that were successfully used for the examples. For patents, the two most consistent in terms of URL performance, text quality and structure extraction numbers, were Free Patents Online (FPO) and EPO Espacenet [7,10]. For journal articles we used the Open Access subset of European PubMed Central and PubMed for abstracts. Performance between operating system and browser alternatives can be configuration-dependent but this evaluation was done on a standard Windows 7 machine using Firefox and Chrome (but note an extension to the Safari browser can chemicalize.org web pages on-the-fly [11]). Features of the standard Microsoft Office suite that proved useful included (a) Notepad for format-stripping and editing text, (b) the ability to transfer either complete URL content, or specific sections to Word and save a PDF for upload to chemicalize.org, and (c) working across multiple windows (e.g. a converted URL open in one, cross-pasting to the chemicalize.org text conversion box in a second and having the a Google interface in a third).

Conversion success rates in CNER are dependent on text quality. For this reason, results from the direct processing of URLs can often be improved by removing confounding formatting. This can be done by converting text sections into a fresh PDF or selected individual IUPAC names for iterative editing via the front page text box. For example, from a 50,000-word patent URL, just the 5,000-word section that encompasses relevant IUPAC names for data-linked examples can be saved as a PDF that will convert rapidly and cleanly on upload. The download options have different utilities. The SDF file can be used as an archive to generate other formats. Alternatively, SMILES produces the smallest file size for batch uploads to databases and are convenient for merging and intersecting result sets from multiple extractions.

### Structure searching

The first questions to answer for extracted structures are their identity or similarity to other sources. For any individual compound the most efficient first-pass is a Google search with the inner skeleton layer of the InChIKey [12]. This will instantly record which major databases include a matching record. For similarity searches, the logical order is chemicalize.org itself, followed by internal, public or commercial databases. For bulk checking, an identity search against PubChem will be exemplified here. To enhance result interpretation a series of MyNCBI custom filters were set up for this work. Two of these were unions for the patent and literature-derived compound records from sources described in the introduction. Two others record the total and unique matches to chemicalize.org (see below). The fourth filter was adapted from the constitutive Rule-of-five parameters astpre-set in PubChem, by the addition of a 250 to 800 Mw window. This provided a useful separation of reagents and intermediates from lead-like compounds exemplified in patents.

One of the reasons for choosing PubChem for triage is that structures from the chemicalize.org result archive have recently been deposited (Source name = chemicalize.org by ChemAxon) [13]. This provides not only the pre-computed relationships of each structure to the neighbor space in PubChem but also the connectivity to all other PubChem sources and “back out” (via chemicalize.org) to user-submitted URLs or documents. As of April 2013 the chemicalize.org source was linked to 297,083 compound identifiers (CIDs). Of these, just over 20% were unique (i.e. the exact structures were not present in other PubChem sources). The fact that 80% of the structures are independently supported by other submissions (according to the CID merging rules) indicates the quality of the chemicalize.org archive. While users need to be aware that the presence in PubChem introduces circularity in terms of structure searches *per se*, any URLs for the chemicalize.org source link via the substance identifiers (SIDs) may have been updated since the deposition date. Thus, not only can the original links in the chemicalize.org entry (accessed via the SID) be different to those chosen by a new user but clicking on any link automatically re-extracts the structures from that URL.

### Batch search in PubChem

For batch searches the PubChem query upload interface is shown below [14].

**Figure 2:**
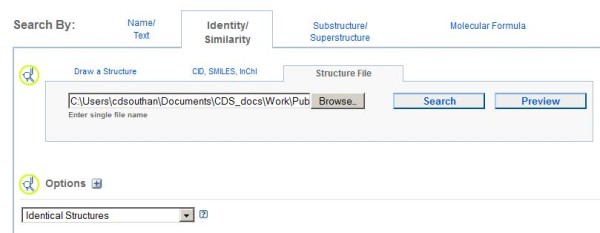
**PubChem Batch Search interface **[14].

By selecting “Structure File” in the interface (Figure 2) a local SMILES, SDF or InChI file from a document extraction can first be uploaded via “Preview” which counts the conversions that conform to the PubChem query input processing rules. The “Identical Structures” default search is recommended to avoid hit-expansions that may cause time-outs. Both the pre-search and post-search filtering options in PubChem are extensive but selected examples of the latter are shown below.

## Results

To present a coherent and relevant set of extractions from a potentially wide range of examples we have made three restrictions. The first is that we have orientated searches around a specific medicinal chemistry theme of Dipeptidyl Peptidase 4 (DPPIV) inhibitors [15]. As a declared drug target for diabetes since 1998, this shows useful specificity as a document keyword search. This extends forwards to current drug discovery research, as well as backwards over many years to patent filings, clinical trials and approved drugs (e.g. saxyglyptin in Figure 1). This protease target therefore makes a good example to explore global R&D activities. The second restriction was to illustrate utility for just the three key document types of patents, papers and abstracts. The third was to orientate results towards answering practical questions that typically arise in the context of a drug discovery project. A set of these is listed below. The first six are document-centric while the last three are structure-centric.

1. Can chemical structures be identified in this document?

2. How many can be extracted?

3. Which ones have database entries?

4. Which database entries have links to this document?

5. Where in the document are the structures specified?

6. Can SAR data be linked to structures in the document?

7. What other documents include this structure?

8. Which database records for this structure have links to other documents?

9. What additional connections can be made using similarity searches?

**Figure 3:**
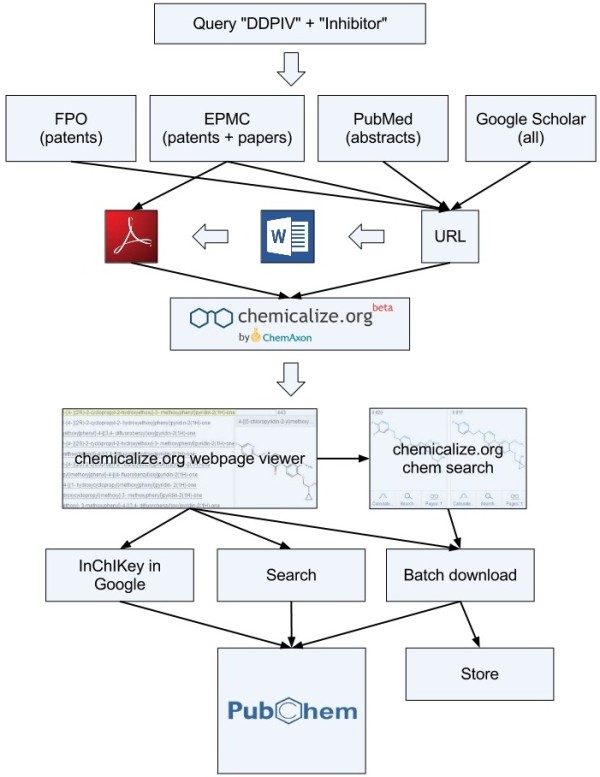
**Workflow Overview.** The diagram covers a representative triage from search to the analysis and storage of results. Note that for simplicity not all options are shown. (Abbreviations used; Free Patents Online = FPO, Europe Pub Med Central = EPMC).

In the context of these restrictions we can present a schematic of the options that can be used in conjunction with chemicalize.org (Figure 3).

### Patent extraction

**Figure 4:**
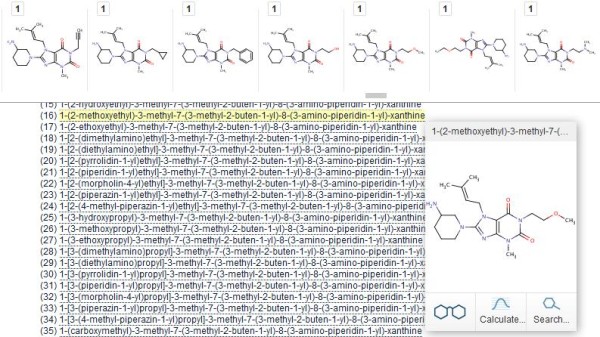
**A converted section from US20120040982.** This shows one of the example series from page 10 in the patent PDF. The details of “example 16” converted from the IUPAC name are highlighted yellow in the list and linked to the grey bar in the structure display ribbon.

To answer the initial questions a recently published DPPIV patent was selected (US20120040982 [16]). The FPO URL was used to extract the entire document via the Webpage Viewer (Figure 4).

**Figure 5:**
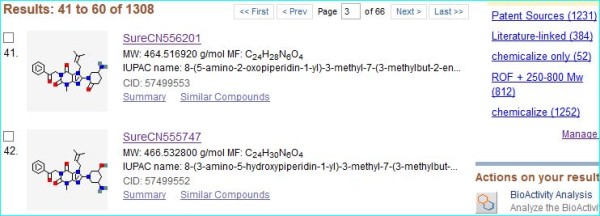
**PubChem structure matches for US20120040982 extraction.** The screenshot is the result from the batch search upload of 1414 SMILES via extraction of the FPO URL for US20120040982. The display of 1308 matches was ranked by CID number and two exemplified structures are shown. The counts matching the five MyNCBI filters set up for this work are highlighted in blue (top right corner).

The question “can chemical structures be identified in this document” was answered by scrolling through a set of examples in the description section (Figure 4). After this qualitative indication of successful conversions, the second question “how many structures were extracted?” had already been answered in top-right hand of the display ribbon as 1414. Scrolling through the web pages indicated that most IUPAC names had been fully converted. Importantly, these included the majority of the structures specified in the main example section (starting at page 46 in the patent). The question “which ones have database entries” was answered by a batch upload query described above (Figure 2). In this way intersects can be generated for any database(s) but the results for PubChem are shown in Figure 5.

The question “do any of these have links to this document?” was answered for individual records by inspecting sources, for example, CID 57499553 (at the top of Figure 5) has two. The chemicalize.org entry (SID 137228062) links to the most recent URLs extracted by users for US20120040982. The SureChemOpen entry (SID 152667195) links to the same document but with 17 additional members of the patent family. The question as to “where in any document an individual database structure is located?” is easiest to answer in reverse by establishing the PubChem match for a chemicalize.org conversion at a certain position. For a patent there are two alternatives, following the chemicalize.org ribbon display in sequence in the document or searching the structure in SureChemOpen (directly on the SureChem website, or via the PubChem link). In this case, SID 152667195 was located to example 496, but note that structures can be specified multiple times in a patent.

**Figure 6:**
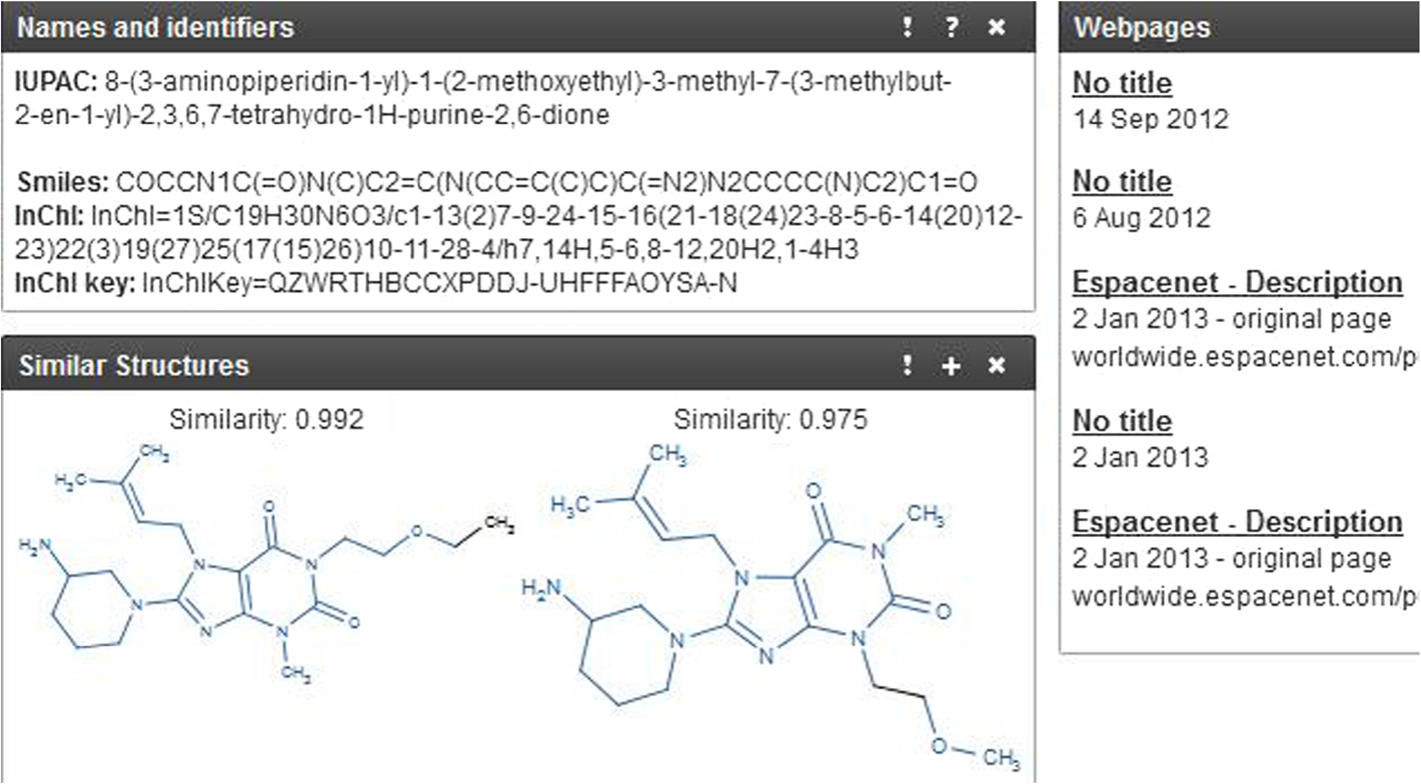
**Selected details from the chemicalize.org display page for “example 16” from US20120040982.** The four “Names and identifiers" are computed. The “Webpages” result is for exact matches in web pages and documents submitted by chemicalize.org users. “Similar Structures” shows the top results of a similarity search against the chemicalize.org archive (in this case two analogues from the same patent).

The question “can SAR data be connected to structures in the document?” requires the prior location of activity results. The table on page 13 of the US20120040982 PDF assigns a DPPIV IC50 of 78 nM to “example 16”. We can then locate this structure by searching the example number in the text. There are many options in the properties display page, from which we have selected three (Figure 6).

The InChIKey was Google-negative but matched CID 57498937 by a direct search in PubChem. This had been submitted by both SureChem (SID 152666516) and chemicalize.org (SID 137227422). The result thus provided an answer to “what other documents include this structure?” as negative. We then addressed the related question “which (other) database records have links to documents?” However, the difficulty associated with this is the substantial amount of common chemistry typically extracted along with the examples. The solution in this case was to prepare a PDF containing only the 38 IUPACs specifically claimed on page 63 of the original document. This was extracted and the results uploaded to PubChem, thereby excluding reagents and prior-art descriptions. This gave 34 CID matches, 32 of which had both SureChem and chemicalize.org as sources. In addition, eight of these had ChEMBL as a third source, thus providing links to journal articles and assay data. For example, CID 24750280, the eighth structure in the claim list, had a published IC50 displayed in the PubChem record for the inhibition of DPPIV in Caco-2 cells of 88 nM derived from PMID 18052023 [17].

Having made total and sectional extractions from this document (a moderate size of patent at 65 pages) benchmarking statistics can be generated (Table 1).

**Table 1:** Extraction, upload and PubChem match statistics for US20120040982

**Extraction source**		**Upload**	**Conv.**	**Fail**	**CIDs**	**SID:CID**	**CZ in PC**	**CZ-only**
Full-text URL	n/a	1414	1364	34	1308	63	1252	52
Main examples (PDF)	497	486	468	0	462	2.1	457	16
Claims-only (PDF)	38	34	34	0	30	3.1	28	0

The row results are derived from; 1) the FPO URL as shown in Figure 5 2) the main example section IUPAC names pasted out of “Description”, saved as a PDF and subsequently uploaded for document extraction, 3) the IUPAC names in the “Claims” section, similarly saved as a PDF for uploaded . The column results are; 1) the count of SMILES downloaded from chemicalize.org and subsequently uploaded, 2) successful PubChem conversions, 3) PubChem conversion failures, 4) PubChem CID exact matches, 5) substance: compound counts (i.e. the average SID: CID ratio), 6) matches in PubChem that included chemicalize.org as one of the CID sources and 6) with chemicalize.org as the only source.

In a typical medicinal chemistry patent it would be difficult to determine the potentially extractable total (row 1, column 1) because of extensive redundancy in the form of repeated exemplifications, reagents, intermediates and Markush components. Notwithstanding, across the first row, columns 1 and 2 show 96% of the uploaded SMILES were verified by the PubChem structure checker. From columns 3 and 5, we can establish that 96% of the extracted structures were already in PubChem. The high SID: CID ratio indicates that these included a substantial amount of common chemistry or known drug structures (i.e. each structure having on average 63 submitters) most of which were already represented in the 1252 chemicalise.org entries. Inspection of the 52 structures unique to this source (column 8) shows lead-like structures that were absent from PubChem. Row 2 provides a direct in-verses-out assessment from the IUPAC names (497) and the conversions by PubChem (468) with a 94% yield. In this row the SID: CID ratio drops to 2.1 because most of these lead-like examples were novel structures from (on average) only two sources, SureChemOpen and chemicalize.org. The claimed compounds (row 3) have an 89% conversion. The 30 matching structures in PubChem have more sources because some of them have been extracted from published papers (i.e. the SIDs include five ChEMBL entries).

The exploration of chemistry in a patent document can have different domain-specific utilities but the basic outcomes can be reviewed. Chemicalize.org has converted the majority of example structures that circumscribe the subject matter and enable further analysis. The observation that most of these were already in PubChem is likely to be the default case for patents from the major authorities, unless they have been published since the latest SureChemOpen deposition. The extensive matches to chemicalize.org confirmed, via the links, that a user had already converted this document (i.e. one of the authors C.S.). The partitioning of novel structures from common chemistry was achieved by isolating the example sections. The SAR-to-structure mapping was discerned by two routes. The first was intra-document via the data in the patent (which could easily be extended to complete the whole table). The second was inter-document connections to published results for some of the same compounds in medicinal chemistry journals (via ChEMBL). The option of sectioning the document provides flexibility, for example in being able to separate structures extractable from the introduction, description, synthetic schema or claims.

### Extraction from papers

**Figure 7:**
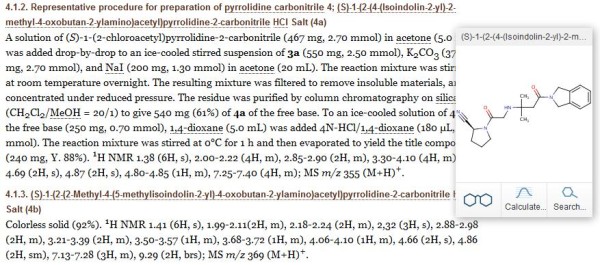
**Extracting the “Methods” section of PMC3305890.** The result for 4a is shown as the topmost IUPAC name.

While the questions we can ask are similar to those for patents, journal papers have the advantages of a smaller document size and typically include explicit specification of the key structures being studied with their bioactivity results. The example chosen was a DPPIV medicinal chemistry paper from European PubMed Central (PMC3305890) with free full text-access published by the company Kenkyusho in 2006 [18]. The question “how many structures could be extracted?” was answered as 52 from the PubMed Central URL. However, the focus of the paper was on just 12 compounds linked to SAR data. Identification of the first example (4a) is shown in Figure 7.

The answer to the question “which extractions have database entries?” was all of them, (i.e. 52 matches in PubChem). The common chemistry was partitioned by making a PDF of just the 12 key IUPAC names. The downloaded structures could then be aligned with the IC50, clearance and P540 metabolism results (Table 1 in PMC3305890) thus answering the question “which data can be connected to which structures in the document?”. The structure search in PubChem included 12 CID matches, all of which had chemicalize.org as a source (here again, this was because the paper had been extracted during this work). Unusually, 11 were unique to chemicalize.org since only one CID 10383508; (4a from Figure 7) had other source links. These thus answered the question “which database records for this structure have links to other documents?”. The SureChemOpen entry, via a same-connectivity link (SID 157613372) established a link to example 61 in a Kenkyusho patent, WO2004067509 [19]. The ChEMBL entry (SID 103476839) was linked to a publication (PMID 16392822) where the IC50 inhibitory activity of this structure against DPPIV was 49 nM [20]. We also used CID 10383508 to answer the question “what additional connections can be made for a structure using similarity searches?” in two ways. The first was to launch “ChemSearch” from the chemicalize.org entry which records 1934 structures out to a Tanimoto similarity score of 0.5. The second was launching the equivalent search within PubChem that recorded 632 matches with a similarity score of 0.85.

Outcomes for this paper extraction included extensive PubChem matches. In addition, all the key compounds were aligned against the SAR table and links to additional relevant documents established. The triaging of similarity results and their connectivity cannot be expanded on here but note that the 2D and 3D neighbor spaces in PubChem can be both explored and graphically clustered for any CID. This extraction also provides a remediation example. The initial conversion pass for the synthetic intermediate in section 4.1.2 from Figure 7 in the paper, failed. Copying this out of the manuscript web page as text string and pasting it into chemicalize.org corrected this to (2S)-1-(2-chloroacetyl) pyrrolidine-2-carbonitrile. This was then InChIKey matched via Google to PubChem CID 11073883 which had 24 sources.

### Abstracts

**Figure 8:**
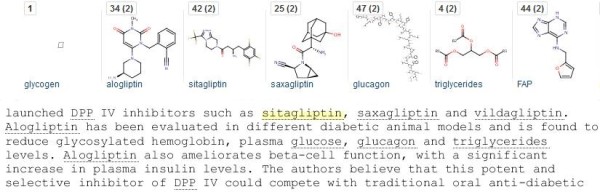
**Result section from a bulk abstract extraction.** The upper part of the figure is a section of the structure display ribbon in order of occurrence in the PDF. Saxaglyptin is highlighted (yellow) from the abstract text for PMID 22651127 (Preclinical development of dipeptidyl peptidase IV inhibitor alogliptin). Recognized chemical terms underlined in text are linked to structures in the ribbon, five of which are shown.

These have the advantage of standardized text “chunks” for extraction but the disadvantage that only a small proportion of the chemical content of a paper may be specified in the abstract. This section describes a bulk result set from PubMed queries. Combining a set of filters (chemistry journals AND “dpp iv OR dipeptidyl peptidase AND inhibitor [Title/Abstract]” AND the last 10 years) produced 295 abstracts. These were downloaded as MEDLINE format because this converts more structures from MeSH annotations than selecting just the abstract text. The first question is “how many structures can be extracted from this set”. The answer, after conversion to a PDF, was 528 structures. A converted section from this document is shown in Figure 8.

The answer to the question “how many have database matches” is 463 but, because the literature-linked filter only recorded 374, the chemicalize.org results provide 89 new abstract-to-database links and 154 structures that are absent in PubChem. Inspection of the ribbon display shows additional utility where, as expected from the query, DPPIV drugs are frequently mentioned. Not only can these be explicitly counted but they can be “stepped through” to locate each occurrence in a specific abstract (e.g. each of the 42 mentions of sitaglyptin). However it should also be noted that Figure 8 includes a false-positive structure. DPP was recognized as synonym for di-n-pentylphthalate because of the spacing in the abstract text (i.e. DPP IV vs. DPPIV).

This analysis indicates useful complementarity with PubMed where chemicalize.org can recognize (and count) structures in abstract sets that are either missing from PubChem or have no direct PubMed-PubChem links. An example is the MeSH supplementary concept IUPAC from PMID 20128619 in the abstract set [21]. This novel DPPIV inhibitor has now become CID 60206521 via the chemicalize.org-only submission (i.e. added to PubChem as a consequence of user extraction and includes a link to the abstract). The simple triage used above (PubMed > result file > chemicalize.org) can be applied to any slice of the 22 million MEDLINE abstracts.

### Intersecting extractions

**Figure 9:**
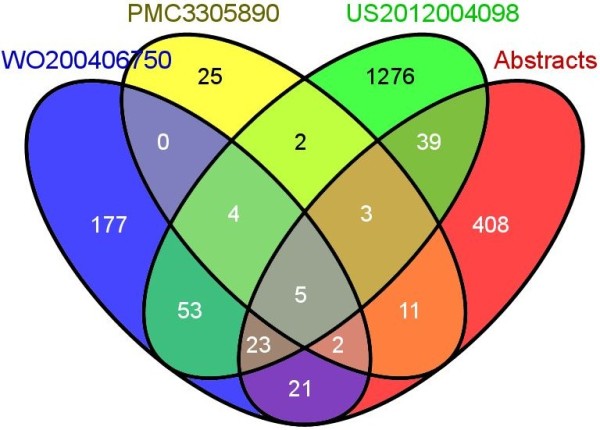
**Venn intersects from extracted documents.** The SMILES input totals (left to right) were 285 for the Kenkyusho patent WO200406750, 52 for the Kenkyusho paper PMC3305890, 1414 for the Boehringer patent US2012004098 and 512 for the DPPIV abstract set.

The examples above have approached connectivity and content questions via database searches. However, it is also important to be able to independently address the question “what other documents include (or omit) this structure?” (e.g. compounds-in-common and/or unique content). This becomes important where the database matches are only partial for any document. There are many technical options for performing the requisite set intersections but the Venny tool provides a useful 4-way overlap [22]. This is demonstrated by using four of the DPPIV-related document extractions described above, namely two patents, one paper and the abstract set. The Venny result is shown in Figure 9.

Details of these intersects and differences need not be expanded here, but the combination of chemicalize.org and Venny allows these to be followed-up. Each subset can be isolated and checked by PubChem searching and/or re-extraction. Not only can any combination of extractions or database lists be used (e.g. SMILES, InChI or even standardized IUPAC names), but Venny can also merge and de-duplicate concatenated sets of chemicalize.org outputs (e.g. in this case the four sets added up to 2,049 unique SMILES).

### Limitations

The ability of chemicalize.org to recognize all structures specified in a section of text, as well as the potential addition of false-positives, is subject to the constitutive limitations of CNER. While some document-specific failure rates are shown in Table 1, it should also be noted that IUPAC names not correctly extracted on a first-pass, can potentially be remediated by simple fixes. One of these, pasting minimally formatted text blocks into “clean” PDFs, has already been mentioned. Another is that common IUPAC name conversion errors (e.g. 1 vs. L, spaces, line breaks, missing brackets or author errors in the primary text) can, in many cases, be iteratively corrected using the front page text input box. False-positives are mainly derived from split IUPAC names, homonym clashes (e.g. some gene symbols being identical to chemical acronyms) unresolvable synonyms (e.g. the same names being used in the literature to refer to different structures) and contextual ambiguity (e.g.” x derivatives” being translated to a structure for “x”). While the chemicalize. org dictionary is subject to regular updates and expansions to reduce these limitations, it should be noted that, in practice, the low level of false-positives are easily spotted and would not usually confound further analysis.

## Conclusions

The results above demonstrate the value of chemicalize.org to answer questions related to the chemical content of the key document types for biomedical research. It should also be pointed out that the strong growth in open-access journal content (as PDF and/or URLs) will expand chemicalization.org options. The approaches outlined are not only technically straightforward for those unfamiliar with cheminformatics but they can also be extended to any text source, including internal documents and chemical information on the web. The ability to make reciprocal document-to-document, document-to-database or document-to-web connectivity is of crucial importance. In addition, the indexing of PubChem and ChemSpider by Google has become complementary and transformative because over 50 million aggregated databases entries (including the chemicalize.org archive) can now be checked for an InChIKey match [12]. Similarity searches in databases extend this connectivity even further.

In regard to connectivity, the absolute correctness and completeness extraction of any document extraction *per se* is less important than the ability to make joins using just a sample of the important extracted structures. For example, there is high value in establishing that patent A, journal article B, PubMed abstract C and database record D, are, from the bioactivity standpoint, probably referring to the same canonical chemical entity. This means that the associated data can be merged between documents, regardless of salt form or isomer differences. In the case of boutique sources that either cross-reference thinly or not at all, chemicalize.org may be the only way to make such joins. There are also synergies with other open tools (in addition to Venny). These include OPSIN for IUPAC names [23], OSRA for chemical images [24] and Utopia for bio-entity recognition [25]. The performance and scope of chemicalize.org is being continually developed, including implementation for mobile phones [26]. It is therefore destined to make an increasing impact in medicinal chemistry, chemical biology and other bioactive chemistry domains.

## Availability and requirements

The application is publicly available for on-line use at chemicalize.org and can be accessed with any modern web browser. There is also a free Android mobile app. The core underlying functionality is available as the ChemAxon's “Naming” commercial product suite that includes the chemical name conversion and mining engine. Use of these products on chemicalize.org is covered by Creative Commons BY-NC-SA 3.0 license.

## Competing interests

CS declares no competing interests. AS is an employee of ChemAxon.

## Authors’ contributions

CS and AS conceived the idea and outline of the paper. AS was senior developer for the chemicalize.org project and CS wrote the manuscript. Both authors read and approved the final manuscript.
